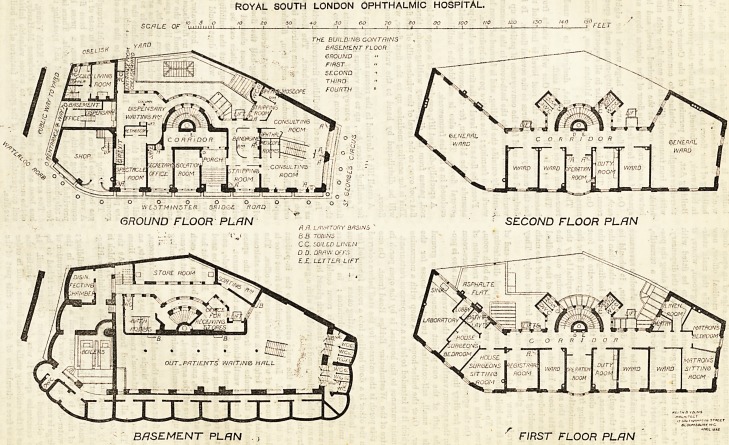# The Royal South London Ophthalmic Hospital

**Published:** 1892-04-30

**Authors:** 


### HOSPITAL CONSTRUCTION.

### THE ROYAL SOUTH LONDON OPHTHALMIC HOSPITAL.

This hospital, which for many years occupied small and most inconvenient premises in St. George's Circus, S.E., has recently been rebuilt, and the new premises are fast approaching completion. The site, which includes that of the old building, is about four times the area formerly occupied, and has frontages to St. George's Circus, Westminster Bridge Road, and Waterloo Road.

The planning of the new building is necessarily rather complicated, and will be best understood by following with the aid of the plans the course of an out-patient from entrance to exit, and then taking the in-patient department separately.

Before doing this it is necessary to point out that one main principle in the design is that the out-patient department must be entirely separate from the in-patient part; and another essential rule governing the arrangement of the out-patient department is that patients, many of whom are blind or semi-blind, must be guided as much as possible from one point to another, and must in no case cross each other or retrace their steps.

To begin, therefore, with the out-patient work: the entrance for out-patients is on the ground floor, or rather pavement level, at the Waterloo Road end. Passing up a passage-way, screened off from the public entrance to Obelisk Yard by a wire-work partition, the patient finds himself in a narrow passage, on the right hand side of which, close to the entrance, is a window in the partition enclosing the office. At this window he obtains his letter, and then passes down a staircase, and, turning to the right again, finds himself in the waiting hall. In all the staircases and passages the width is kept sufficiently small that a person can touch each side if a passage, or, if a staircase, can hold the hand-rail on each side, and so guide himself along. The waiting-hall is not yet permanently seated, but will be provided with seats so arranged that the patients must pass one way only. At the further end of the hall are four sets of water-closets, one set each for old patients, male and female, and one set each for new patients, male and female. In each case the entrance is by a door opening outwards only, and the return by a door opening inwards only, and as the closets are some feet below the hall floor, handrails for the patients' guidance are provided.

The double staircase shown near the end of the waiting-hall, gives access to the ground floor. One side leads up to the north consulting-room, the other to the south consulting-room. The patients, therefore, are at the foot of the stairs, divided into two streams, one stream going to one medical officer, the other stream to the other. Each consulting-room is lighted by a large semi-circular-headed window, filled with one sheet of plate-glass. For the use of each medical officer there are two small recesses formed with slate partitions, for ophthalmoscopic work, and an examining room, and, accessible from both consulting rooms, is a bandaging-room. Here, it should be noted, that a patient enters the examining-room by one door and leaves by another, and the same with the bandaging-room, and that the doors in each case have been carefully arranged, so that they open with the stream of patients, and in no case can a patient going in

FOURTH FLOOR PLAN

A A LAVATORY BASINS

C.C SOILED LINEN

D D DRAW-OFFS

E.E. LETTER LIFT

THIRD FLOOR PLAN

ROYAL SOUTH LONDON OPHTHALMIC HOSPITAL.

the proper direction impinge upon the edge of an open door. A patient having been seen by the medical officer, now passes along the curved passage-way outside the main staircase of the hospital to a smaller waiting-room, where, if necessary, he goes into the spectacle-room to be fitted with glasses, or wait his turn to get his medicine at the dispensary. From thence he passes down the staircase and along the passage under part of the spectacle-room, through a turnstile, and out into the street.

In order to make clear the point about absolute severance between the out and in-patient departments, it should be noted that the only access to the waiting-hall, other than the patients' entrance, is by way of two doors leading into the open yard, one close by the lift, the other opposite the foot of steps leading from Obelisk Yard. On the ground floor the only communication with the rest of the hospital is by the door close to the south examining room opening on to the porch. This door is intended for the use of the medical staff. The windows shown along the carved wall of the main staircase looking into the passage are for light only. They are of iron and glass, and are fixed.

Returning now to the basement, at the Waterloo end of the site is the boiler house, engineer's shop, and disinfecting chamber. To the north of the waiting-hall is a bath-room, with two baths and a w.c., and office for the reception of stores. On the opposite side of the yard is a large store-room and a room for dirty linen. Ample storage room also is provided in the spacious vaults under the pavement.

To the left of the out-patient entrance, on the ground floor, is a small, self-contained, two-storey house for the engineer. Over the boiler-house is a shop with a mezzanine floor, which it is intended to let as a temperance restaurant.

The main entrance to the hospital is by the open porch in the centre of the Westminster Bridge Road front. On one side of this porch is the entrance for the staff before referred to. On the other is a door leading into a room intended for use as an isolation room for separating cases of infectious disease until fetched away by ambulance; in the rare occurrence of the death of a patient this room could also be used as a mortuary. It is entirely lined with glazed bricks and tiles, has an asphalte floor, and is provided with a gas furnace for destroying fæces.

Facing the visitor is the main entrance to the in-patient portion of the hospital. Immediately inside the front door is a corridor, at one end of which is the Secretary's office. The semi-circular block of brickwork around which the staircase winds contains the smoke flue from the boilers, the space around which forms a ventilation flue from the basement.

The staircase is planned in the form of a semi-circle, and within the two enclosing walls are two flights, one to be used exclusively for ascent, the other for descent. Each flight has a handrail at each side, and at the top and bottom of each flight swing bars will be arranged, which will prevent anyone from going up the descending staircase or down the ascending one.

The first floor contains the resident House Surgeon's rooms, Registrar's room, three single wards for paying patients, an operation-room, the Matron's room, pantry, and the linen store. These all are entered from the corridor. At the Waterloo Road end of the building is a laboratory, which is entered by crossing the flat roof over the medicine waiting-room and the engineer's quarters.

Two small wings project from the staircase building; in one of these is a water-closet and a slop sink; in the other a water-closet and a shower bath. The construction of these wings is somewhat novel, and sanction for it had to be specially obtained from the London County Council. In order to economise space it was desirable to restrict the thickness of the enclosing walls to nine inches; and in order to obtain as free a circulation of air about this part of the building, it was further desired to keep the height of the w.c.'s to the necessary minimum of seven feet. With a building of four floors in height, it was impossible to do this in brickwork, or, indeed, at all under the provisions of the Building Act. The permission of the County Council was therefore sought and obtained to construct these buildings with an iron framework filled in at each floor with nine-inch brickwork roofed over at the required level, and leaving between each roof and the floor of the closets above an open space of some four feet.

On the second floor are two large wards for eight beds each, three single bed wards, a duty-room, and an operation-room. The third floor is similar in all respects.

On the fourth floor are the kitchen offices, and rooms for nurses and servants.

The roof is an asphalte flat, designed to be used as a recreation space for patients and staff.

There are in all five lifts. One which starts from the basement and finishes at the roof level is intended mainly for the conveyance of stores to the several floors. It is also designed to be used for the conveyance of patients up to the wards, or from thence to the bath-room in the basement. It is entirely outside the building, and is formed of open lattice-work iron framing; the motive power is hydraulic.

From the flat at the level of the first floor a hand-power lift ascends to the kitchen, for the conveyance of meals to the several floors. This also is outside the building, and is enclosed in a lattice framework.

A specially-devised lift, consisting of a cage working on a rod, passes from the dispensary waiting-room, through the flat roof over, to the level of the window-sills of the first floor. This is for sending medicine up to the first floor, whence it will be distributed by hand to the various rooms on all floors.

A small lift is devised in the recess marked E on the plan, for sending letters, &c., up to the various floors.

Lastly, a lift for the conveyance of instruments will be fixed from the operation-room on the second floor to that on the third floor.

In the construction of the building fireproof materials have, as far as possible, been used throughout. The floors are of iron joists embedded in concrete, and the staircase is of concrete. The roof is of concrete and iron, finished with asphalte.

The walls throughout the out-patient department are lined with glazed bricks. In the waiting-hall the floor is of asphalte, and is laid to slope to an open channel covered with an iron grating, so that the whole place can be flushed out with a hose. All angles throughout the building are rounded, and ledges or places on which dust could lodge have been so far as possible avoided.

The building is warmed throughout by hot water, and except in the engineer's house, there are no fireplaces anywhere. The coils have been specially designed for the work, and are made with a view to occupying as little space as possible. They are cased in with sheet iron covers, which are made easily removable, and in which the angles are all rounded. Fresh air is brought in from the outside to the coil casing, and the openings in the latter admit the fresh warmed air into the rooms. In all rooms extraction shafts are placed, their areas varying according to the size of the rooms.

The building is being fitted with electric light throughout, gas being also laid on to certain parts for use in case of need. A very complete system of electric bells has been fitted, communicating with a central indicator, a press being provided at the head of each bed in the wards. There is also a telephone with central switch board and speaking tubes for communication along short distances.

The architect for the building is Mr. Keith Young, who has been assisted throughout by Professor McHardy, one of the surgeons to the hospital, to whose valuable initiative and co-operation most of the special features of the building are due.

## Figures and Tables

**Figure f1:**
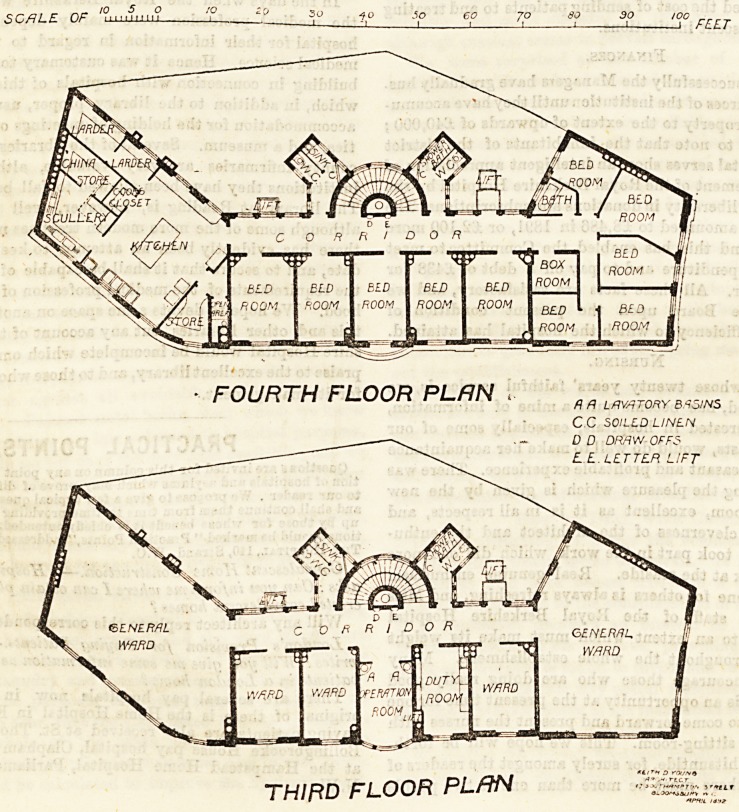


**Figure f2:**